# Outcomes of complex Descemet Stripping Endothelial Keratoplasty performed by cornea fellows

**DOI:** 10.1186/s12886-018-0946-4

**Published:** 2018-10-30

**Authors:** Jacquelyn Daubert, Terrence P. O’Brien, Eldad Adler, Oriel Spierer

**Affiliations:** 1 Bascom Palmer Eye Institute, University of Miami Miller School of Medicine, Palm Beach Gardens, FL 33418 USA; 2 Department of Ophthalmology, Wolfson Medical Center, Sackler Faculty of Medicine, Tel Aviv University, Tel Aviv, Israel

**Keywords:** Cornea fellows, Descemet Stripping Endothelial Keratoplasty, DSEK, Ophthalmology fellowship training

## Abstract

**Background:**

A major obstacle that academic institutions face is the steep learning curve for cornea fellows initially learning to perform Descemet Stripping Endothelial Keratoplasty (DSEK). The purpose of this study is to evaluate the outcomes of complex DSEK performed by cornea fellow supervised by an attending surgeon at an academic institution.

**Methods:**

Patients who underwent a complex DSEK procedure performed by a cornea fellow during the years 2009-2013 were included. All the surgeries were supervised by the same cornea attending. All patients had a minimum follow-up of 6 months. Charts were reviewed for demographic data, intraoperative and postoperative complications and clinical outcomes. Corneal graft survival was calculated using the Kaplan-Meier analysis.

**Results:**

Fifty-seven eyes of 55 patients (mean age 77.5 ± 8.5 years) were included in the study with a mean follow-up time of 16.4 ± 15.6 months. Previous graft failure, presence of a tube and history of trabeculectomy were the leading diagnoses to define the surgery as complex. No intraoperative complications occurred. In 21.1% of cases a corneal graft detachment was documented in the first postoperative day. Mean visual acuity improved from 1.06 LogMAR (20/230) preoperatively to 0.39 LogMAR (20/50, *p* < 0.001) by the sixth postoperative month and to 0.52 LogMAR (20/65, *p* < 0.001) at the last follow-up visit. Graft failure rate was 29.8%. Kaplan-Meier analysis found a 67.2% graft survival rate at 20 months.

**Conclusions:**

Complex DSEK can be performed successfully with an acceptable postoperative complication rate by cornea fellows during their training period when supervised by an experienced attending.

## Background

Descemet Stripping Endothelial Keratoplasty (DSEK) has been adopted worldwide as an alternative to penetrating keratoplasty in corneal pathologies restricted to the endothelium such as Fuchs endothelial dystrophy and pseudophakic bullous keratopathy. In these cases, DSEK has shown overall better results than penetrating keratoplasty [1] including a faster visual recovery, decreased astigmatic change, and reduced risk of suture related complications [2, 3].

DSEK has its limitations in complex cases such as in the presence of previous tube shunt procedures, trabeculectomies and previously failed DSEK or penetrating keratoplasty grafts [4–8]. For patients with tube shunts, the tube placement in the anterior chamber may interfere with the surgical placement of the corneal graft and allow air to escape causing possible graft detachment in the first postoperative day [4]. In DSEK performed on patients with tube shunts, the tube needs to be revised to provide enough room for the donor graft in the anterior chamber and to prevent future trauma to the endothelium [4]. Early graft detachment is also a concern in trabeculectomies, as the air can leak through the sclerotomy [5]. Patients with trabeculectomies and tube shunts usually have advanced optic nerve glaucomatous damage. An elevation in intraocular pressure after DSEK surgery or during the procedure with the air bubble injection could cause loss of vision in these susceptible patients [5]. DSEK performed after a failed penetrating keratoplasty or failed DSEK is a technically challenging procedure and tends to have higher rates of postoperative complications [4, 6, 7].

There have been several studies evaluating resident and fellow outcomes during various ophthalmology surgical procedures [9]. When it comes to cataract surgery, fellows and residents have a higher complication rate as compared to the attending surgeons [10–12]. The major obstacle that academic institutions face is the steep learning curve for cornea fellows initially learning to perform DSEK [13]. Hashemi et al. concluded that DSEK can be performed successfully by cornea fellows in their training period with acceptable outcomes [14]. In the United States only one study, done by Chen et al., evaluated DSEK outcomes when performed by fellows as compared to an attending surgeon. The authors found no difference in visual outcome or endothelial cell loss between DSEK done by an experienced cornea surgeon and cornea fellow under the supervision of an attending surgeon [1]. They raised the question, however, of could these results be partially due to the attending surgeons choosing less complex cases for the fellows [1].

The purpose of this study is to report the outcomes of complex DSEK performed by cornea fellows supervised by an attending surgeon at an academic institution.

## Methods

A retrospective chart review was performed of patients who underwent a complex DSEK procedure from January 2009 to November 2013 and was performed by a cornea fellow under the supervision of one cornea attending (T.P.O.) at the Bascom Palmer Eye Institute. The DSEK procedure was defined as complex if the patient had a functioning tube shunt or filtering bleb, presence of silicone oil in the anterior chamber, presence of vitreous in the anterior chamber, presence of peripheral anterior synechiae or iridocorneal adhesions, history of failed penetrating keratoplasty or failed DSEK, a history of angle closure glaucoma with the presence of a shallow anterior chamber or poor visualization necessitating the use of trypan blue during the DSEK procedure. Patients with less than 6 months of follow up were excluded. In this case series, patients in whom graft failure occurred in the first 6 months following surgery remained included in the study and were continued to be followed up. The medical charts were reviewed for demographic data, indication for DSEK, concurrent procedures, intraoperative and postoperative complications and clinical outcomes. Pre- and postoperative measurements included Snellen best-corrected visual acuity and intraocular pressure measured by TONO-PEN (Medtronic, Doral, Florida, USA). When reporting visual acuity results, patients were divided into those with no other significant ocular pathology and to those with other ocular pathology limiting vision including previous retinal detachment involving the macula, chronic cystoid macular edema, proliferative diabetic retinopathy, advanced glaucoma and non-arteritic ischemic optic neuropathy. Postoperative data at 6 months and at final visit was included. Graft failure was defined as an edematous cornea with failure to maintain deturgescence lasting beyond a period of 1 month of intense corticosteroid therapy or vascularization and scarring resulting in irreversible loss of central graft clarity.

### Surgical procedure

Prior to the surgery patients received a peribulbar block with intravenous sedation. The cornea was marked with an 8.25 mm manual trephine. Two 1 mm paracenteses tracts were made at the limbus and viscoelastic was injected into the anterior chamber. A 2.2 mm temporal beveled corneal incision was fashioned at the limbus. Using a reverse Sinskey hook (Bausch & Lomb, San Dimas, CA) the Descemet’s membrane and endothelium were scored in a circular fashion and then stripped off with an endothelial stripper (Katena, Denville, NJ). Trypan blue was not used routinely to stain the endothelium. The Viscoelastic was removed from the anterior chamber using the irrigation/aspiration unit. The temporal incision was enlarged to 4.2 mm. The donor corneal tissue was trephined to 8.25-mm, separated from the donor stroma and placed into the anterior chamber. The graft was unfolded and balanced salt solution was used to form the anterior. The temporal wound was sutured with two 10-0 nylon sutures. The anterior chamber was completely filled with air to force the apposition of the graft to the recipient stromal bed and the patient was left face up for 10 min. A fluid-air exchange was then performed and using a 32-gauge needle air was injected to fill about 80% of the anterior chamber. At the end of the surgery, a topical cycloplegic drop was applied. The patient was then instructed to lie in the face-up position until the following day. Antibiotic and corticosteroid drops were applied after surgery and were tapered accordingly. Patients were examined 1 day, 1 week and 1 month after surgery and after that as needed.

### Statistical analysis

Data was recorded on Microsoft Excel Software version 14.1 (Microsoft Corp., Redmond, WA, USA). Snellen best-corrected visual acuities were converted to logarithm of the minimal angle of resolution units (LogMAR) to allow for averaging and statistical analysis. BCVA and IOP before and after the surgery were compared using a t-test. Time to graft failure was determined using the Kaplan-Meier analysis log rank test (XLSTAT software, Addinsoft Inc., Brooklyn, NY, USA). A *p*-value < 0.05 was considered statistically significant. Data is presented as means (± standard deviation).

## Results

Fifty-seven eyes of 55 patients (56.4% female, 52.6% right eye) were included in this study. The mean age of the patients was 77.5 ± 8.5 years (range: 49-91 years). Mean follow-up time was 16.4 ± 15.6 months (range: 6-60 months). The main indication for DSEK was a history of failed DSEK or penetrating keratoplasty graft. Indications for DSEK are presented in Table 1. All eyes had a posterior chamber intraocular lens, with 6 eyes (10.5%) having scleral fixated intraocular lens. Table 2 summarizes the criteria for designating the DSEK as complex for the 57 eyes in this study. Fifteen eyes (26.3%) had more than one of these criteria: 11 eyes (19.3%) had two criteria and four eyes (7.0%) had 3 criteria. Concurrent surgical procedures during the DSEK are summarized in Table 3. No intraoperative complications were documented in any of the cases.

**Table 1:** Indications for Descemet Stripping Endothelial Keratopalsty (*n* = 57 eyes)

Indication	N (%)
Previously failed graft	33 (57.9)
Pseudophakic bullous keratopathy	22 (38.7)
Fuchs endothelial dystrophy	1 (1.8)
Silicone oil keratopathy	1 (1.8)

**Table 2:** Criteria for designating Descemet Stripping Endothelial Keratoplasty as Complex (*n* = 57 eyes)

Cause	N (%)
Previous corneal graft failure	33 (57.9)
Presence of tube shunt	16 (28.1)
Previous trabeculectomy	13 (22.8)
Vitreous prolapse	4 (7.0)
peripheral anterior synechiae/iridocorneal adhesions	4 (7.0)
Shallow anterior chamber or angle closure glaucoma	3 (5.3)
Poor visualization necessitated trypan blue	2 (3.5)
Silicone oil in the anterior chamber	1 (1.8)

The total percent adds up to more than 100 because some patients had two or three criteria defining the Descemet Stripping Endothelial Keratoplasty as complex

**Table 3:** Concurrent Surgical Procedures performed during the Descemet Stripping Endothelial Keratoplasty (*n* = 57 eyes)

Concurrent surgical procedure	N (%)
Tube revision/trimming	4 (7.0)
Anterior vitrectomy	4 (7.0)
Synechiolysis	2 (3.5)
Silicon oil removal from anterior chamber	1 (1.8)
Epithelial debridement	1 (1.8)
Tube revision/trimming + anterior vitrectomy + pupilloplasty	1 (1.8)
Anterior vitrectomy + synechiolysis	1 (1.8)

### Visual acuity and intraocular pressure

Mean preoperative best-corrected visual acuity in all eyes was 1.06 LogMAR (20/230, range: 20/50 to hand motions). Mean best-corrected visual acuity improved to 0.39 LogMAR (20/50, range: 20/20 to 20/800, *p* < 0.001) by the sixth postoperative month and to 0.52 LogMAR (20/65, range: 20/25 to hand motions, *p* < 0.001) at the last follow-up visit. Twenty-two eyes (38.6%) had ocular pathology limiting vision beside the corneal pathology, including 8 cases (14%) of advanced glaucoma, 8 cases (14%) with chronic cystoid macular edema, 4 cases (7%) of previous retinal detachment, 1 case (1.8%) with proliferative diabetic retinopathy and 1 case (1.8%) of non-arteritic ischemic optic neuropathy. After excluding these patients, mean preoperative best-corrected visual acuity was 1.12 LogMAR (20/263, range: 20/70 to hand motions), which improved to 0.36 LogMAR (20/46, range: 20/20 to 20/800, *p* < 0.001) by the sixth postoperative month and to 0.48 LogMAR (20/60, range: 20/25 to hand motions, p < 0.001) at the last follow-up visit.

When compared to preoperative measures, mean intraocular pressure did not change 6 months and at last follow up: 14.1 ± 6.6 mmHg (range: 3 to 36 mmHg), 13.2 ± 6 mmHg (range: 3 to 31 mmHg, *p* = 0.55) and 13.3 ± 5 mmHg (range: 2 to 25 mmHg, *p* = 0.38), respectively.

### Postoperative complications and graft failure

Postoperative complications were observed in 19 eyes (33.3%). Graft detachment occurred in 12 eyes (21.1%). Nine eyes (15.8%) were managed by one rebubbling, 2 (3.5%) required 2 rebubblings and 1 (1.8%) required 3 rebubblings. The lenticule reattached successfully in 9 cases. In the other 3 patients, the cornea never cleared after the surgery and 2 patients underwent another corneal transplantation. One patient refused additional intervention. IOP higher than 30 mmHg was seen in 6 patients (10.5%, range: 32 to 53 mmHg) on the first day after the surgery. Three had pupillary block and were treated with anterior chamber paracentesis. Suture abscess occurred in 1 patient (1.8%) 20 days after the surgery. Although prompt treatment with topical antimicrobials, the DSEK graft eventually failed. There were no cases of endophthalmitis.

**Fig. 1:**
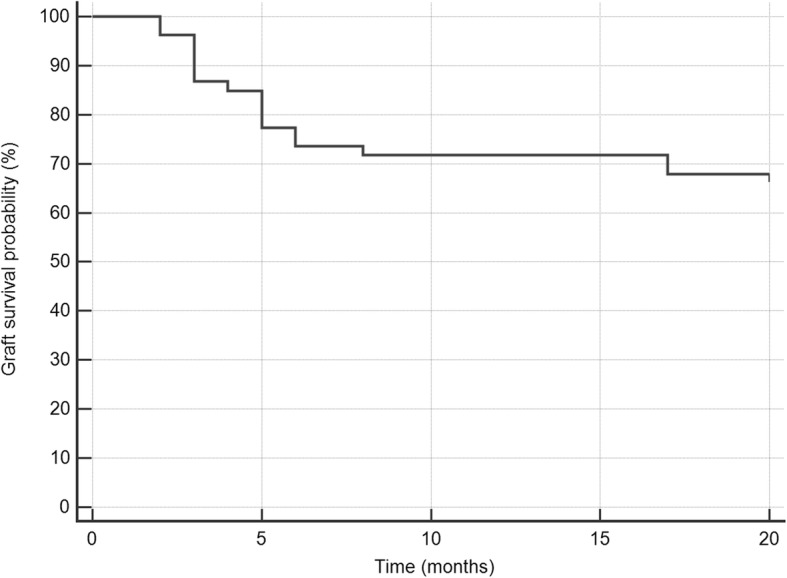
Kaplan-Meier analysis showing the time to graft failure. At 2 months, 2 grafts failed. At 3 months, 5 grafts failed. At 4 months, 1 graft failed. At 5 months, 4 grafts failed. At 6 months, 2 grafts failed. At 8 months, 1 graft failed. At 17 months, 2 grafts failed

Secondary graft failure was observed in 17 cases (29.8%) and on average occurred 5.71 ± 4.54 months after surgery (range: 2 to 17 months). All of these patients had a previously failed corneal graft, a previous glaucoma surgery, or both. Five of these cases had a graft detachment on the first postoperative day necessitating rebubbling. Kaplan-Meier analysis found a 67.2% graft survival rate at 20 months (Fig. 1). No graft rejection episodes were documented.

## Discussion

To our knowledge this is the first study to report the outcomes of complex DSEK performed by cornea fellows under the supervision of one experienced surgeon. In this study, mean best-corrected visual acuity improved significantly after the surgery. Postoperative complications were observed in 33.3% of cases and mainly included early graft detachments and spikes in intraocular pressure. Secondary graft failure was observed in 29.8% of the cases.

DSEK is more challenging and with higher rates of postoperative complications in complex eyes [4]. Previous glaucoma surgery is one of the significant risk factors for graft failure [8, 15–17]. It is associated with at least a 4-fold increase in the risk of failure when accounting for other factors [15, 18, 19]. Ward et al. assessed the association of glaucoma treatment with graft survival after penetrating keratoplasty and DSEK. Out of 156 DSEK-operated eyes, the 5-year Kaplan-Meier graft survival was higher than 90% in eyes without any glaucoma surgery or with only medical glaucoma treatment, and as low as 50% in eyes with surgical intervention for glaucoma [17]. Iverson et al. reported that 76.9% of the grafts in eyes with prior trabeculectomy failed by 36 months [8], while Price et al. found that 60% failed by 5 years [16]. In our study, half of the patients had a history of a tube shunt or trabeculectomy. The Kaplan-Meier analysis showed a 67.2% graft survival rate at 20 months, which is in line with previous studies.

DSEK performed after a failed penetrating keratoplasty or failed DSEK can be technically more difficult, and tends to have higher rates of postoperative complications [4, 6, 7]. There is a risk that remnants of the friable Descemet membrane will be left behind leading to poor attachment of the new lenticule [7]. In DSEK performed after a failed penetrating keratoplasty with a glaucoma drainage device, the graft dislocation rate was reported by Clements et al. to be 67% [6]. This may be attributed to the compounded complexity of the glaucoma drainage device with the failed penetrating keratoplasty [6]. When looking at outcomes of DSEK after a failed penetrating keratoplasty, graft dislocations rates of 14- 43% have been reported [6, 7, 20]. In the present study, the majority of the cases were complex due to previous graft failure or the presence of a glaucoma drainage device. The graft dislocation rate was 21.1%, which is comparable to the published rate in the literature. Secondary graft failure rate was 29.8% (17 out of 57 eyes), which is on the low side compared to the range of 15.9% to 76.9% found in the literature for complex eyes [8, 15–17, 21].

Attention in the literature has been directed towards the steep learning curve of endothelial keratoplasty procedures among experienced cornea surgeons [13]. Several authors reported relatively high donor dislocation rates in the early stages of their endothelial keratoplasty learning curve ranging from 25 to 27% [13, 22]. They also reported primary graft failure rates ranging from 7 to 11% in these cases [22, 23]. Excessive tissue handling by an inexperienced DSEK surgeon increases the endothelial trauma leading to an increased rate of postoperative complications [24, 25]. One study looked at the first 50 cases of novel DSEK surgeons compared with their second set of 50 cases and found that rates of primary failure, secondary graft failure and lenticule dislocation decreased as the surgeon gained experience with the procedure. Graft dislocation rates decreased from 20 to 10% between the early and late groups [24]. Terry claimed that the most important step for a successful DSEK is a strict surgical technique, regardless of a surgeon’s experience [26]. In the present study, graft detachment occurred in 21.1% of cases. This rate is in agreement with the above studies. The graft dislocation rate was expected to be higher since all of our cases were complex and all were performed by inexperienced surgeons. The supervision of an experienced cornea attending and adherence to a strict surgical technique played a major role in our study’s good results.

One of the biggest challenges academic institutions face is balancing the training of fellows with the risk of higher postoperative complications for their patients and possible poorer outcomes [1]. Only two studies have reported the outcomes of fellows performing non-complex DSEK. One, executed by Chen et al., was conducted in the United States and compared the outcomes of supervised fellows and attending surgeons. They found no significant difference between cornea fellows and attending surgeons in terms of visual acuity and postoperative complications [1]. Mean postoperative best-corrected visual acuity was 20/36 in the fellow cases. They also reported a very low graft dislocation rate (1%) which is much lower than the 21.1% dislocation rate we had. Nevertheless, their study population included mainly simple (non-complex) cases [1], as opposed to our study population consisting solely of complex cases. The second study done by Hashemi et al. at Tehran evaluated the performance of fellows mainly in non-complex DSEK cases without a control group. They reported a lenticule detachment rate of 21.8%, and a graft failure rate of 10.2% [14], results that are comparable to ours. They reported a mean postoperative best-corrected visual acuity of 20/118 at the 6-month follow-up visit [14]. Our 6 month mean postoperative best-corrected visual acuity was 20/50 which is slightly lower than the 20/36 reported by Chen et al. but better than the 20/118 in Hashemi et al. study [1, 14].

Limitations of this study include its retrospective nature and the relatedly small number of patients. In addition, consistent data regarding the patient’s endothelial cell count throughout follow up was not available, so comparison to the preoperative endothelial cell count was not done.

## Conclusions

For cornea pathology involving the endothelium, there has been an increasing trend over the last years from penetrating keratoplasty towards DSEK, offering a faster visual recovery with small refractive change [8]. Nevertheless, graft survival rates have not changed dramatically [8] and a substantial number of patients still require recurrent corneal grafting. It is estimated that 80 million people will have glaucoma by 2020 [27]. Academic institutions, as tertiary referral centers, are facing an enlarging number of patients with failed DSEK or penetrating ketaoplasty and patients with a history of glaucoma surgery who are now candidates for DSEK. These institutions face the challenge of training upcoming cornea surgeons while being a referral center for a large number of complex cases, causing their fellows to perform a rising number of complex cases. According to this study, complex DSEK can be performed successfully with an acceptable postoperative complication rate and outcomes by cornea fellows during their training period when supervised by an experienced attending.

## Abbreviation

DSEK: Descemet Stripping Endothelial Keratoplasty
